# Sex Role Segregation and Mixing among Men Who Have Sex with Men: Implications for Biomedical HIV Prevention Interventions

**DOI:** 10.1371/journal.pone.0070043

**Published:** 2013-08-01

**Authors:** Benjamin Armbruster, Sourya Roy, Abhinav Kapur, John A. Schneider

**Affiliations:** 1 Department of Industrial Engineering and Management Sciences, Northwestern University, Evanston, Illinois, United States of America; 2 Department of Electrical Engineering and Computer Science, Northwestern University, Evanston, Illinois, United States of America; 3 Pritzker School of Medicine, University of Chicago, Chicago, Illinois, United States of America; 4 Departments of Medicine and Health Studies, University of Chicago, Chicago, Illinois, United States of America; University of Washington, United States of America

## Abstract

**Objective:**

Men who have sex with men (MSM) practice role segregation – insertive or receptive only sex positions instead of a versatile role - in several international settings where candidate biomedical HIV prevention interventions (e.g., circumcision, anal microbicide) will be tested. The effects of these position-specific interventions on HIV incidence are modeled.

**Materials and Methods:**

We developed a deterministic compartmental model to predict HIV incidence among Indian MSM using data from 2003–2010. The model’s sex mixing matrix was derived from network data of Indian MSM (n = 4604). Our model captures changing distribution of sex roles over time. We modeled microbicide and circumcision efficacy on trials with heterosexuals.

**Results:**

Increasing numbers of versatile MSM resulted in little change in HIV incidence over 20 years. Anal microbicides and circumcision would decrease the HIV prevalence at 10 years from 15.6% to 12.9% and 12.7% respectively. Anal microbicides would provide similar protection to circumcision at the population level despite lower modeled efficacy (54% and 60% risk reduction, respectively). Combination of the interventions were additive: in 5 years, the reduction in HIV prevalence of the combination (−3.2%) is almost the sum of their individual reductions in HIV prevalence (−1.8% and −1.7%).

**Conclusions:**

MSM sex role segregation and mixing, unlike changes in the sex role distribution, may be important for evaluating HIV prevention interventions in international settings. Synergies between some position-specific prevention interventions such as circumcision and anal microbicides warrant further study.

## Background

Unique to men who have sex with men (MSM) in many settings internationally (e.g., India and Peru) is the adoption of a fixed sexual role (insertive or receptive) rather than a versatile role during intercourse. This role segregation is important because of the different transmission risks of insertive and receptive anal sex and because biomedical interventions can potentially target specific individuals based on their sex role (insertive or receptive). Some candidate prevention interventions for MSM, currently under study such as circumcision and anal microbicides would likely only protect insertive and receptive partners respectively [1], [2]. Others such as condoms and antiretroviral based treatments protect MSM irrespective of their sex position [3]. The goal of this study is to model the effect of these position sensitive interventions on HIV prevalence over a 20-year period using sex role data from South India.

In heterosexuals, the effectiveness of circumcision and vaginal microbicides in preventing HIV is dependent upon sex position. For example, female receptive partners of circumcised men are not protected [4] and to date, there is no evidence that the male partners of women who utilize microbicides are protected. Additionally, in most Western settings, there has been little evidence for the benefits of circumcision among MSM [5] even stratifying for sex role and focusing on insertive-only sex participants [6]. In international contexts, however, there is theoretical and observational support for circumcision as protective among MSM who only assume an insertive role [7]. Thus, sex role is critically important in developing and implementing position-specific interventions.

Previous mathematical models have found that individual sex role preference and mixing by sex role has a strong contribution to HIV transmission dynamics among MSM [8]–[10]. For example, Goodreau and colleagues concluded that a population of MSM with complete role versatility would have twice the prevalence of HIV over time compared to a population of MSM with role segregation [8]. However, preferential mixing among versatile MSM did not change the overall population prevalence but affected which individuals became infected. Most existing models of HIV transmission consider a group of susceptibles that may become infected; some models separate the susceptible population into a few groups such as male, insertive-MSM, etc.; and seldom do models consider a sexual network [11], [12]. The more detailed the model, the more it is able to predict the distributional impact of HIV and to some extent the more accurate its predictions. Our model is between the latter two types: we use sexual network data to parameterize mixing in a compartmental model where we distinguish among different sex roles.

## Methods

### Study Population

Our study population consists of MSM participating in behavioral and sexual network surveys between 2003 and 2010 in Andhra Pradesh, India [13]–[16]. Participants were recruited in Hyderabad the capital of Andhra Pradesh state (population of ∼85 million), which has the highest numbers of HIV infected in India [17]. The surveys all took place in what are known as “hot-spots” and affiliated drop-in centers managed by partnering community based organizations (CBO). These settings, are generally public venues such as bus stops or parks that incorporate higher risk segments of the MSM community, including sex workers and their clients, many of whom are married [18], [19], who engage in transactional sex in the hot-spot venues and receive HIV prevention services in the drop-in centers. All procedures and protocols in the principal studies were approved by the relevant institutional review boards in the United States and India.

### Model Development

A compartmental deterministic model is used to predict the spread of HIV among Indian MSM over 20 years. This model is based on that presented in Goodreau et al. [8] The population is divided into six compartments based on sex roles (insertive, receptive and versatile) and infection status (HIV−, HIV+). Members of the population are assumed to enter the population with a sex role, possibly become infected, and finally exit the population (due to AIDS or other causes). Our model has three refinements on the previous model. First, we consider condom use. Second, we do not assume proportional mixing but instead make use of data on the sexual mixing among different roles (Goodreau et al. considered assortativity, which is one dimension of sexual mixing, for a part of their paper). Proportional mixing or “random” mixing is when each versatile MSM has equal chances of partnering with any other MSM in the network. Third, we consider the changing distribution of sexual roles. Our model does this by changing the distribution of sexual roles among new members entering the population or by allowing individuals to change their role over time. The model is represented by the equations in Figure S1 and the full set of parameters is listed in Table 1. For comparison, we also build a simple SI model with homogenous mixing depicted in Figure S2. SI model is a Susceptible Infected compartmental model which utilized differential equations.

**Table 1:** Parameters for model of HIV transmission among men who have sex with men in Southern India.

Parameter	Value				Reference
Initial population in various roles, *n_i_*(0), *n_r_*(0), *n_v_*(0)	56.6%, 19.3%, 24.1%				[15], [16]
Initial fraction of HIV+ among various roles, *n_i+_*(0)/*n_i_*(0), *n_r+_*(0)/*n_r_*(0), *n_v+_*(0)/*n_v_*(0)	9.6%, 25.2%, 24.7%				[14]–[16]
Number of uninfected individuals added every day to population pool, μ	1.2				[22]
Condom usage probability per sexual encounter, c	0.629				[15], [16]
Probability that condom usage prevents transmission, α	0.9				[35]
Annual mortality rate for HIV− and HIV+ individuals, γ−, γ+	1/46, 1/15				[23]–[25], [36]
Probability of becoming infected per sexual encounter by role, *β_i_*, *β_r_*, *β_v_*	0.0016, 0.0048, 0.0032				(see discussion)
Distribution of roles in entering population, *µ_i_, µ_r_, µ_v_*	*n_i_, n_r_, n_v_*				[13], [14]
Exception: scenario 2 (disproportionate inflow)	29%, 9%, 62%				(see discussion)
Annual probability of changing roles from *x* to *y*, *f_xy_*	0				
Exception: scenario 3, *f_iv_, f_rv_* (role change)	2.3%, 2.3%				(see discussion)
Number of sexual interactions an individual of role *x* has with a member of role *y* in a month (sexual mixing), *P_xy_*	*P*	i	r	V	[15], [16]
	i	0.00	2.10	0.13	
	r	13.02	0.00	0.52	
	v	4.96	2.27	1.68	

### Sexual Mixing Data

We used data from 2010 network survey of MSM (n = 4604) [15], [16] to specify the initial distribution of sex roles, *n_x_,* and to derive the sexual mixing matrix *P* = [*P_xy_*]. Here *P_xy_* is the number of sexual interactions an individual of role *x* has with a member of role *y* in a month. We ignored reports of insertive-insertive and receptive-receptive encounters because of the limited number of them and the difficulty in interpreting them with the existing data. There is no epidemiological data determining a threshold for when a MSM should be considered versatile nor is there biological significance for various thresholds in terms of HIV transmission. For this analysis, network members were categorized as versatile if they self-identified as versatile, were described as “versatile” by a study respondent, or if the ratio of insertive vs. receptive categorizations of the network member was between 3∶1 and 1∶3. The number of sexual encounters between individuals in role *x* with individuals in role *y* can be estimated as either *P_xy_* times the number of individuals in role *x* or *P_yx_* times the number of individuals in role *y*. So that our equations in Figure S1 are consistent (i.e., balanced) we take the average of the two estimates.

We consider four scenarios. The first is a status quo scenario that serves as a reference. In the baseline scenario, individuals do not change their sex roles (the *f_xy_* are zero); the distribution of sex roles among individuals entering the population, *µ_x_*, equals that of the initial population; and the observed mixing matrix [*P_xy_*] is used. In the second and third scenarios, the fraction of versatiles increases over time. This is an ongoing trend in India documented by ethnographic work [20] and behavioral surveys spanning 2003–2010 [13], [14]. In the second scenario, this is modeled by assuming that the individuals entering the population are disproportionally versatile. In this scenario, the distribution of sex roles among individuals entering the population is set to match the rate of increase in the versatile fraction of the population observed between 2003 [13] (18.0%, Dandona personal communication) and 2008 [14] (25.2%). Our choices lead to the fraction of versatiles increasing from 24.1% initially to 31.3% in five years. In the third scenario, this is modeled by assuming that individuals change their roles to versatile over time (i.e., practice change). In this scenario, we set the rate of role changes to match the increase in the versatile population over ten years seen in scenario two. The fourth scenario considers proportional mixing to judge the value of the sexual mixing data in *P*. In this scenario we do not change the average number of partners individuals have in each role (i.e., the row sums remain the same), we only change how the roles of these partners are distributed. We assume that the roles of the partners are distributed in proportion to their number in the population. Thus the modified mixing matrix changes over time. For example, receptives have 13.54 interactions per month; the ratio of insertives to versatiles is 56.6∶24.1 at time 0; hence under proportional mixing, the number of receptive-insertive encounters per month, *P_ri_*, is 13.54*56.6/(56.6+24.1) = 9.50 in the modified mixing matrix at time 0. Again, we also assume that *P_ii_* = *P_rr_ = *0 and take the average of the two estimates to determine the rate of sexual encounters between different roles. Details are in Figure S1.

### Other Parameters

We used data from 2010 [15], [16] to specify the condom usage probability, *c*. The initial HIV prevalence in each role is estimated by combining data from our field surveys [14]–[16]. Condoms are assumed to prevent transmission α = 90% of the time [21]. We start with an initial population of 10,000 adults, and we model population growth by adding new HIV− individuals to the population at a rate of 1.2 per day. This leads to a total population of 10,146 in one year and 10,690 in five years, approximately matching the 10,141 and 10,725, respectively, that one would expect from India’s annual population growth rate of 1.41% [22]. The mortality rate *γ_-_* for HIV− individuals implies a life expectancy of 46 years to reflect the 48.3 and 43.8 year life expectancy of male Indians age 20–24 and 25–29, respectively [23]. For HIV+ individuals, *γ*_+_ implies a life expectancy of 15 years to reflect the 9.5 year [24] or 11 year [25] median survival time without treatment; the 23–28% (2010 guidelines) and 36–55% (2006 guidelines) treatment coverage [26]; and the 6 to 15 year gain from treatment [27]. The transmission probabilities per encounter varies from population to population and may depend on many factors including the length of the encounter, whether the insertive partner withdraws before ejaculation, etc. Thus we set these probabilities by first fixing their relation to another and then varying their magnitude so that the baseline prevalence trajectory is roughly flat, matching existing data [28]. We assume that *β_v_* = (*β_i_+β_r_*)/2 since versatile individuals take both insertive and receptive roles. Assuming that 30% of insertives are circumcised and the transmission probabilities among MSM [29], we find that the ratio of the receptive to insertive transmission probabilities, *β_r_*/*β_i_*, is 3.

### Biomedical HIV Prevention Intervention

We simulate the effects of biomedical HIV prevention interventions that are currently under investigation for MSM [1], [2]. Except for PrEP (pre-exposure prophylaxis- anti-retroviral therapy taken to prevent HIV acquisition) [3], these candidate interventions will likely have efficacies contingent upon a specific sex role. Currently, there are no efficacy data on a microbicide or circumcision in the prevention of HIV acquisition in receptive and insertive only MSM sex roles respectively. However, we do have compelling data on HIV acquisition from heterosexual insertive and receptive sex role positions with these two interventions and utilize a range of efficacy values from these trials (Table 2) [30], [31]. We use these estimates as a starting point for the efficacy of circumcision and a hypothetical anal microbicide in preventing HIV acquisition for MSM by sex role. We also use these estimates because the suggested higher rates of anal microbicide adherence that might be expected among a MSM population that already uses anal lubricants, such as in India, could offset any potential lower efficacy when compared to vaginal microbicides [1]. The assumed risk reduction of each intervention is applied to *β_r_* and/or *β_i_* (depending on the affected sexual role(s)), with *β_v_* again the average of the *β_r_* and *β_i_*.

**Table 2:** One-way sensitivity analysis of various scenarios on HIV prevalence among South Indian men who have sex with men (changes are in percentage points).

Scenario	Prevalence in 5 years	Prevalence in 10 years	Prevalence in 20 years
*Status Quo (Baseline, scenario 1)*	16.0%	15.9%	16.1%
β_v_ = β_i_	15.7% (−0.3%)	15.4% (−0.5%)	15.2% (−0.8%)
β_v_ = β_r_	16.3% (+0.3%)	16.4% (+0.5%)	17.0% (+0.9%)
Role change: *f*_iv_:*f*_rv_ = 3∶1	16.2% (+0.2%)	16.6% (+0.7%)	18.6% (+2.5%)
Role change: *f*_iv_:*f*_rv_ = 1∶3	16.0% (+0.0%)	16.1% (+0.2%)	16.7% (+0.6%)
Condom usage halved	20.2% (+4.2%)	24.5% (+8.6%)	32.8% (+16.7%)
Circumcision (60% rr)	14.3% (−1.6%)	12.7% (−3.2%)	10.2% (−5.9%)
Anal microbicide (38% rr)	14.8% (−1.2%)	13.6% (−2.3%)	11.9% (−4.1%)
(54% rr)	14.2% (−1.7%)	12.6% (−3.2%)	10.3% (−5.8%)
Circumcision+Microbicide (38%)	13.3% (−2.7%)	11.0% (−4.9%)	7.8% (−8.2%)
(54% rr)	12.9% (−3.1%)	10.3% (−5.6%)	6.9% (−9.2%)
PreP (44% rr)	13.5% (−2.5%)	11.4% (−4.5%)	8.4% (−7.6%)
(74% rr)	12.0% (−4.0%)	9.0% (−6.9%)	5.2% (−10.9%)
HIV+ Mortality, γ_+_, Halved	18.3% (+2.4%)	20.8% (+4.9%)	26.4% (+10.3%)

PrEP – pre-exposure prophylaxis; rr - risk reduction.

## Results

### Status Quo Simulation

**Figure 1:**
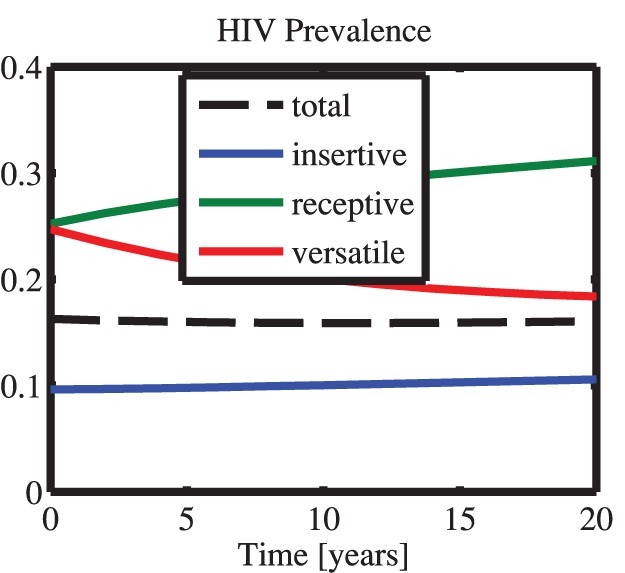
Dynamic HIV prevalence over a 20 year period in Southern India. This model does not account for the various sex-role distribution scenarios described in Table 1: disproportionate inflow (ie increasing proportion of versatiles in the population), role change (insertives or receptives “becoming” versatile), proportional mixing (random mixing; versatile equally likely to partner with any other MSM in population).

Figure 1 shows the fraction of the population in various sex roles, the ratio of the population to that at time zero, and the prevalence over 20 years. The overall HIV prevalence remains approximately constant. Throughout the simulation horizon, the prevalence of receptives is higher than that of versatiles, which in turn is higher than that of insertives.

### Simulations with Changing Community

**Figure 2:**
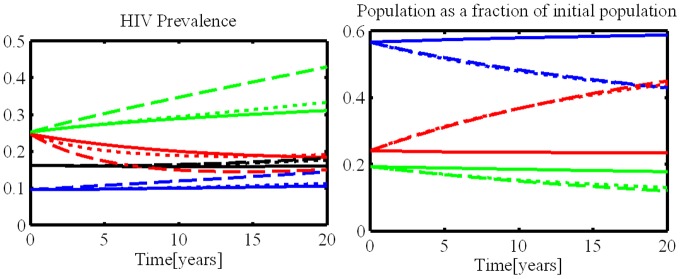
Increasing numbers of versatiles to the model and the resulting changes in HIV prevalence over a 20 year period in Southern India. * *Solid lines are for scenario 1 (status quo), dashed lines for scenario 2 (disproportionate inflow), and dotted lines for scenario 3 (role change). Black is for the total population, blue for insertive, green for receptive, and red for versatile men who have sex with men. Scenario 4 is not shown. X-axis is years of model; y-axis is HIV prevalence.

Figure 2 summarizes the overall prevalence over time for the status quo scenario, the two scenarios that model the increasing fraction of versatiles, and the scenario with proportional mixing. The prevalence in all scenarios is within 0.6 percentage points of each other the first ten years. Over a twenty year horizon, the prevalence in the non-status quo scenarios increases, with the scenario with disproportionally versatile inflow (scenario 2) achieving the highest prevalence (2.1 percentage points above the status quo scenario).

### Sensitivity Analyses

We conducted one-way sensitivity analysis where we start with the baseline (scenario 1; status quo) and vary parameters one at a time to determine their impact on the prevalence in 5, 10, and 20 years (Table 2). The prevalence is insensitive to our choice of β_v_ because this transmission probability applies to only a small fraction of interactions, those between two versatile individuals. Overall we find that at the population level, role change has little effect on overall HIV prevalence. In contrast, halving condom use or halving HIV mortality (from say increased treatment coverage) causes HIV prevalence to grow rapidly. Among biomedical prevention interventions, PrEP has the greatest impact overall on decreasing HIV prevalence with only 12.0% of the population infected in five years. Circumcision and an anal microbicide combined reduce HIV prevalence by an amount that is almost the sum of their individual reductions in prevalence. Figure S3 presents additional sensitivity analyses.

## Discussion

Our model not only provides insights into the growing MSM HIV epidemic in India, but also into several factors that are important for modeling HIV transmission and simulating the potential effects of biomedical HIV prevention among MSM in general. First, the HIV prevalence in this population is 0.3–0.8 percentage points lower under existing sex network mixing patterns than under assumptions of proportionate mixing. Additionally, we find that increasing sex role versatility leads to moderate increases of HIV prevalence over time, with slightly larger increases seen when this increasing versatility is achieved through new versatile MSM joining the population rather than through role change. As might be expected, biomedical interventions that are protective for both receptive and insertive partners such as PrEP, would have the greatest impact on the epidemic, decreasing HIV prevalence to 5.2% in 20 years. This is however, assuming >90% adherence; an adherence rate much higher than seen in the limited data on PrEP acceptability from India [32]. Finally, we find that anal microbicides and circumcision, modeled at HIV prevention efficacy rates in heterosexuals, would have modest effects on the HIV epidemic.

Of note, this is the first model that includes data on MSM sex network mixing where ties are matched (i.e., each respondent reported on sex partnering status with other respondents in the network). The observed sexual mixing is not proportional. Notably, sexual mixing data demonstrated a 50% increase in the number of receptive-insertive encounters per month at time 0 (18,500), when compared to the proportionate mixing model (11,963 encounters per month). Due to the multiple reports provided by the network survey we expect that this reflects the actual mixing rather than for example receptives believing their versatile partner is actually insertive. Observed sex mixing also demonstrates some receptive-receptive and insertive-insertive mixing in this setting, corroborating our own anecdotal observations as well as those reported by others [20]. Comparisons of proportional models across studies is difficult given the higher mixing of versatiles with other versatiles described by Goodreau et al. [8] when compared to our population. Goodreau et al. find that “preferential mixing among versatile MSM does not change overall prevalence” [8]. This agrees with the specific case we consider, where the prevalence estimates from proportional mixing are not much higher than those using the observed (preferential) mixing pattern. This gives us confidence in the accuracy of our model.

The idea of a breakdown in role segregation (i.e., increasing versatiles in the population) as having an effect on HIV prevalence, we argue, is unproven based upon our model. In contrast to other studies which suggest greater rates of HIV at the population level with increasing versatile sex role practice, our data suggest only an insignificant change in HIV prevalence with almost no change in HIV prevalence at 20 years under a disproportionate inflow of versatile MSM assumption or under the assumption that MSM change roles. Suggestions that MSM in India are becoming increasingly versatile, as direct influence from the West is of interest [20], but of little importance to the trajectory of the HIV epidemic. Efforts should be made instead to focus on specific biomedical interventions and how they may fit within traditional sex roles and a potentially increasing versatile sex role.

Our model had several limitations. The parameters were based upon MSM recruited from “hot-spots” and may not reflect the larger and more hidden MSM population in India, many of whom are insertive-only MSM. This would serve to bias our HIV prevalence estimates upwards. However, our sex network data included many clients of receptive-only MSM sex workers demonstrating much higher numbers of insertive-only MSM than previously reported in India. [33] Second, the efficacy rates for microbicide and circumcision were based upon efficacy in heterosexuals. The actual efficacy may or may not be different among MSM. However, our goal was not to compare existing and/or hypothetical prevention interventions across a population, but to demonstrate how these interventions based upon the best available data might work within an HIV epidemic where role segregation and potential increasing versatility are evident. Third, we did not consider the degrees of role versatility of individuals both in terms of proportions of insertive to receptive sex partners as well as event versatility occurring within one sexual encounter. Finally, we did not model treatment or HIV progression in detail or the spread to and interaction with the wider population (e.g., the wives of these MSM).

Public health programmers will need to consider sex role segregation in efforts to promote HIV prevention interventions in many international settings that are likely to have efficacy rates sensitive to the sex role of the end user. Consistent with previous work, the importance of network data for modeling disease spread and forecasting the effect of future interventions that limit the sexual transmission of HIV is critical. Differences in projections exist between models that incorporate sex network mixing and those that do not [8], [34], which could affect downstream recommendations for HIV intervention implementation. If circumcision and anal microbicides are found to be efficacious, their protection may be sex role specific. HIV prevention may need to be reconfigured, with separate modalities targeting each member of a MSM sexual dyad to maximize potential for synergistic effects.

## Supporting Information

Figure S1 (DOCX)

Figure S2 (DOCX)

Figure S3 Sensitivity analysis that vary the distribution of sexual roles in the initial population (top) and among the inflow (bottom). The status quo scenario is labeled SQ. The top contour plot in Figure S3 (3a) demonstrates the final prevalence in 10 years as we vary the initial population fractions. We then varied the inflow fractions in the model. We examine the prevalence in 10 years as we vary the fraction of new individuals in various roles. The two axes of the bottom contour plot (3b) show the fraction of versatile and receptive individuals in the total population in 10 years (points in the shaded region are possible scenarios). We see that in the situations where we end up with the most insertive-only MSM are also those where the HIV prevalence is least. (TIF)
