# Supplementary material for: Sex Role Segregation and Mixing among Men Who Have Sex with Men: Implications for Biomedical HIV Prevention Interventions
Source: PLoS One. 2013 Aug 1;8(8):e70043. doi: 10.1371/journal.pone.0070043 (PMC3731341; doi:10.1371/journal.pone.0070043)
Supplement: Figure S1 [file pone.0070043.s001.docx]

**Figure S1**

$$\frac{dn_{i-}}{dt}=(\mu_{i}-\gamma_{-}n_{i-}-f_{iv}n_{i-})-\frac{n_{i-}}{n_{i}}(1-\alpha c)\beta_{i}\left[N_{iv}\frac{n_{v+}}{n_{v}}+N_{ir}\frac{n_{r+}}{n_{r}}\right]$$

$$\frac{dn_{r-}}{dt}=(\mu_{r}-\gamma_{-}n_{r-}-f_{rv}n_{r-})-\frac{n_{r-}}{n_{r}}(1-\alpha c)\beta_{r}\left[N_{rv}\frac{n_{v+}}{n_{v}}+N_{ri}\frac{n_{i+}}{n_{i}}\right]$$

$$\frac{dn_{v-}}{dt}=(\mu_{v}-\gamma_{-}n_{v-}+f_{iv}n_{i-}+f_{rv}n_{r-})-\frac{n_{v-}}{n_{v}}(1-\alpha c)\left[\beta_{r}N_{vi}\frac{n_{i+}}{n_{i}}+\beta_{i}N_{vr}\frac{n_{r+}}{n_{r}}+\beta_{v}N_{vv}\frac{n_{v+}}{n_{v}}\right]$$

$$\frac{dn_{i+}}{dt}=(-\gamma_{+}n_{i+}-f_{iv}n_{i+})+\frac{n_{i-}}{n_{i}}(1-\alpha c)\beta_{i}\left[N_{iv}\frac{n_{v+}}{n_{v}}+N_{ir}\frac{n_{r+}}{n_{r}}\right]$$

$$\frac{dn_{r+}}{dt}=(-\gamma_{+}n_{r+}-f_{rv}n_{r+})+\frac{n_{r-}}{n_{r}}(1-\alpha c)\beta_{r}\left[N_{rv}\frac{n_{v+}}{n_{v}}+N_{ri}\frac{n_{i+}}{n_{i}}\right]$$

$$\frac{dn_{v+}}{dt}=(-\gamma_{+}n_{v+}+f_{iv}n_{i+}+f_{rv}n_{r+})+\frac{n_{v-}}{n_{v}}(1-\alpha c)\left[\beta_{r}N_{vi}\frac{n_{i+}}{n_{i}}+\beta_{i}N_{vr}\frac{n_{r+}}{n_{r}}+\beta_{v}N_{vv}\frac{n_{v+}}{n_{v}}\right]$$

$N_{xy}=N_{yx}=(n_{x}P_{xy}+n_{y}P_{yx})/2$ for x=i,r,v.

The subscripts *i*, *r*, and *v* denote the insertive, receptive, and versatile roles respectively. Here *n_x-_*, *n_x+_*, and *n_x_ = n_x-_ + n_x+_* are functions of time representing the number of HIV- individuals, the number of HIV+ individuals, and total number of individuals in role *x*. The *N_xy_* are functions of time representing the number of sexual interactions per month between individuals in role *x* and role *y*.

With proportional mixing, we use a modified mixing matrix [*P_xy_*] determined by the distribution of roles, and thus, changing over time:

*P’_ri_=P’_ir_=*(*p_r_* *n_i_*/(*n_i_+n_v_*) + *p_i_ n_r_*/(*n_r_+n_v_*))/2,

*P’_iv_=P’_vi_=*(*p_i_ n_v_*/(*n_r_+n_v_*) + *p_v_ n_i_*/(*n_i_+n_r_+n_v_*))/2

*P’_rv_=P’_vr_=*(*p_r_* n_v_/(n_i_+n_v_) + *p_v_* n_r_/(*n_i_+n_r_+n_v_*))/2

*P’_vv_*= *p_v_ n_v_*/(*n_i_+n_r_+n_v_*)

*P’_ii_*=*P’_rr_*=0

where *p_x_ = ∑_y_* P*_xy_*, ensuring that the number of sexual encounters per month for an individual in role *x*, remains the same as in the original mixing matrix *P*.
