# Supplementary material for: Sex Role Segregation and Mixing among Men Who Have Sex with Men: Implications for Biomedical HIV Prevention Interventions
Source: PLoS One. 2013 Aug 1;8(8):e70043. doi: 10.1371/journal.pone.0070043 (PMC3731341; doi:10.1371/journal.pone.0070043)
Supplement: Figure S2 [file pone.0070043.s002.docx]

**Figure S2**

This is a simple SI model with the contact rate calculated as an average of the contact rates for the three different sexual roles weighted by their initial population fractions.

$$\frac{dn_{-}}{dt}=\mu-\gamma_{-}n_{-}-(1-\alpha c)\beta n_{-}n_{+}/n^{2}$$

$$\frac{dn_{+}}{dt}=\mu-\gamma_{+}n_{+}+(1-\alpha c)\beta n_{-}n_{+}/n^{2}$$

where *μ = μ_i_ + μ_r_ + μ_v_*, *n = n_-_ + n_+_*, and *β = ∑_x,y=i,r,v_ β_x_* (*n_x_*(*0*)*P_xy_+ n_y_*(*0*)*P_yx_*)/2*.* Here (*n_x_*(*0*)*P_xy_+ n_y_*(*0*)*P_yx_*)/2 is N_xy_, our estimate of the number of sexual encounters per month at the beginning of the simulation between individuals of role *x* and role *y*.

One-Way Sensitivity Analysis (Changes are in percentage points)

| **Scenario** | **Prevalence in  5 years** | **Prevalence in  10 years** | **Prevalence in  20 years** |
| --- | --- | --- | --- |
| *Status Quo (Baseline)* | 16.2% | 16.0% | 15.2% |
| β_v_ = β_i_ | 15.5% (-0.7%) | 14.6% (-1.3%) | 12.9% (-2.3%) |
| β_v_ = β_r_ | 16.9% (+0.7%) | 17.4% (+1.4%) | 17.8% (+2.6%) |
| Condom usage halved | 20.7% (+4.5%) | 25.2% (+9.3%) | 34.1% (+18.9%) |
| Circumcision (60% rr) | 15.2% (-1.0%) | 14.1% (-1.8%) | 12.0% (-3.1%) |
| Anal microbicide (38% rr) | 14.5% (-1.7%) | 12.9% (-3.0%) | 10.2% (-5.0%) |
| (54% rr) | 13.9% (-2.3%) | 11.8% (-4.1%) | 8.5% (-6.6%) |
| PreP (44% reduction) | 13.6% (-2.6%) | 11.4% (-4.6%) | 8.0% (-7.2%) |
| (74% reduction) | 12.1% (-4.1%) | 9.0% (-7.0%) | 5.0% (-10.2%) |
| HIV+ Mortality, *γ_+_*, Halved | 18.5% (+2.3%) | 20.6% (+4.6%) | 24.3% (+9.1%) |
