# Supplementary figures and images for: Sex Role Segregation and Mixing among Men Who Have Sex with Men: Implications for Biomedical HIV Prevention Interventions
Source: PLoS One. 2013 Aug 1;8(8):e70043. doi: 10.1371/journal.pone.0070043 (PMC3731341; doi:10.1371/journal.pone.0070043)

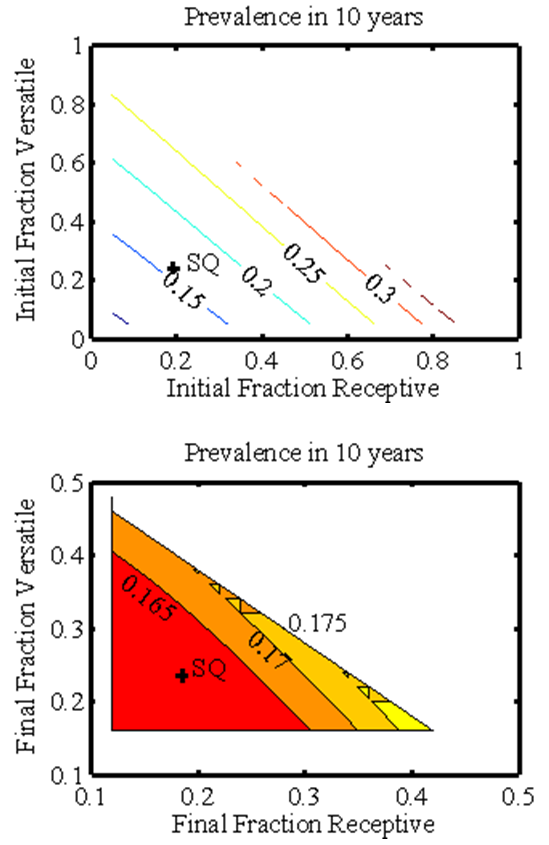

Supplement: Figure S3 — (TIF) [file pone.0070043.s003.tif]
